# Mechanisms of Plasma Ozone and UV-C Sterilization of SARS-CoV-2 Explored through Atomic Force Microscopy

**DOI:** 10.1021/acsami.4c11057

**Published:** 2024-09-06

**Authors:** Jinseung Bae, Petr Bednar, Rong Zhu, Cheolwoo Bong, Moon Soo Bak, Sarah Stainer, Kyoungjun Kim, Junghun Lee, Chulsoo Yoon, Yugyeong Lee, Omobolaji Taye Ojowa, Maximilian Lehner, Peter Hinterdorfer, Daniel Ruzek, Sungsu Park, Yoo Jin Oh

**Affiliations:** 1 School of Mechanical Engineering, Sungkyunkwan University (SKKU), Suwon 16419, Republic of Korea; 2 Veterinary Research Institute, CZ-62100 Brno, Czech Republic; 3 Department of Medical Biology, Faculty of Science, University of South Bohemia, CZ-37005 Ceske Budejovice, Czech Republic; 4 Department of Experimental Biology, Faculty of Science, Masaryk University, CZ-62500 Brno, Czech Republic; 5 Institute of Biophysics, Johannes Kepler University Linz, Linz A-4020, Austria; 6 Samsung Electronics, Suwon 16677, Republic of Korea; 7 Department of Biomedical Engineering, Sungkyunkwan University (SKKU), Suwon 16419, Republic of Korea; 8 Institute of Parasitology, Biology Centre of the Czech Academy of Sciences, CZ-370 05 Ceske Budejovice, Czech Republic

**Keywords:** sterilization mechanisms, infectivity test, topographical characteristics, structural characteristics, binding activity

## Abstract

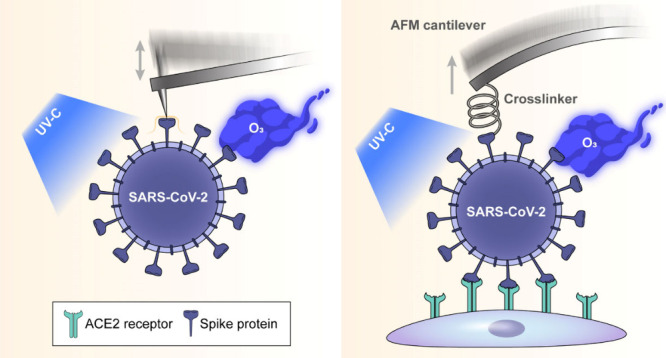

Ultraviolet-C (UV-C) radiation and ozone gas are potential mechanisms employed to inactivate the severe acute respiratory syndrome coronavirus 2 (SARS-CoV-2), each exhibiting distinct molecular-level modalities of action. To elucidate these disparities and deepen our understanding, we delve into the intricacies of SARS-CoV-2 inactivation via UV-C and ozone gas treatments, exploring their distinct molecular-level impacts utilizing a suite of advanced techniques, including biological atomic force microscopy (Bio-AFM) and single virus force spectroscopy (SVFS). Whereas UV-C exhibited no perceivable alterations in virus size or surface topography, ozone gas treatment elucidated pronounced changes in both parameters, intensifying with prolonged exposure. Furthermore, a nuanced difference was observed in virus–host cell binding post-treatment: ozone gas distinctly reduced SARS-CoV-2 binding to host cells, while UV-C maintained the status quo. The results derived from these methodical explorations underscore the pivotal role of advanced Bio-AFM techniques and SVFS in enhancing our understanding of virus inactivation mechanisms, offering invaluable insights for future research and applications in viral contamination mitigation.

## 1 Introduction

Severe acute respiratory syndrome coronavirus 2 (SARS-CoV-2), the virus causing the COVID-19 pandemic, can persist on surfaces for up to a month, underscoring the need for surface disinfection technologies.^1,2^ In this regard, UV-C radiation and ozone gas (O_3_) treatment have been identified as effective methods for virus inactivation. UV-C, with its ability to disrupt the genetic structures of microorganisms, can rapidly inactivate SARS-CoV-2, while ozone gas, which targets and oxidizes microbial surfaces, is another effective method.^3,4^ Significant research (such as that by Lo et al.) has shown that UV-C irradiation at 253 nm can inactivate SARS-CoV-2 within a mere 30 s, without altering its particle morphology.^5^ In contrast, Murata et al. explored varying concentrations of ozone gas treatment and found a notable reduction in SARS-CoV-2 infectivity, though its efficacy is sensitive to humidity levels.^6^ Further, Lee et al. demonstrated that a 10 s exposure to ozone gas at 120 ppm could result in a remarkable 10,000-fold reduction in the infectivity of the human coronavirus (HCoV-229E).^7^ However, these studies have been limited in their capacity to delve into the molecular-level changes in the virus caused by these inactivation methods.

Bio-AFM (atomic force microscopy) has played a fundamental role in examining viruses at a molecular level, offering valuable insights into their structure, susceptibility, and response to various disinfection methods. Multiple Bio-AFM-based studies have elucidated the detailed structure and physical properties of viruses, and the structural changes after disinfectant treatments.^8−11^ Nevertheless, prior investigations using Bio-AFM often evaluated virus samples in air, which can result in sample dehydration, protein denaturation, and potential structural damage. Furthermore, there is a lack of research on how the individual force characteristics of viruses are altered post-treatment with UV-C and ozone gas especially in aqueous environment.

**Figure 1:**
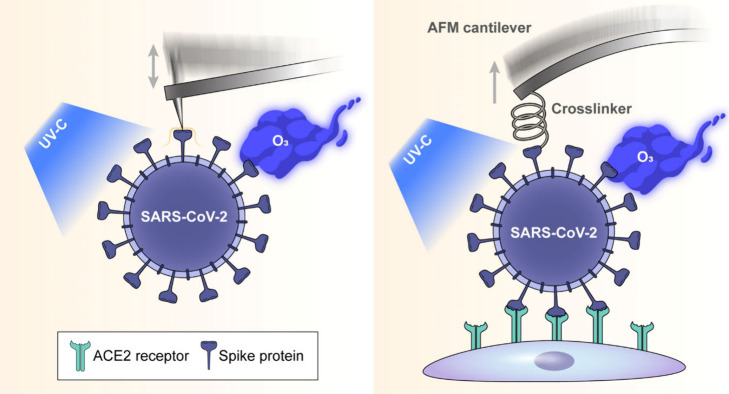
Schematic of Bio-AFM topographic imaging and SVFS methodologies applied to SARS-CoV-2 after UV-C or ozone gas treatment. All measurements were performed under physiological conditions.

To investigate the structural changes of SARS-CoV-2 under different sterilization methods, we utilized Bio-AFM and single virus force spectroscopy (SVFS) in this study (Figure 1). Our experimental design entailed the application of UV-C radiation at 275 nm and ozone gas using a dielectric barrier discharge plasma generator, exposing samples to varying durations of treatment in a solution-based environment.^7^ By using UV-C LEDs instead of the traditional sterilization method of low-pressure mercury lamps (254 nm), we increased sterilization efficiency by implementing a wavelength (275 nm) that is close to the DNA/RNA absorption wave of pathogens. The use of Bio-AFM has enabled a comprehensive exploration of the virus’s topography, providing an essential baseline understanding of its inherent structural characteristics and establishing a benchmark for comparing alterations post-treatment. Additionally, the incorporation of SVFS has allowed for an in-depth analysis of the virus’s binding interactions (notably with Vero E6 cells) under various treatment conditions, presenting a detailed view of the functional implications that structural changes might have on viral binding efficacy.

## 2 Materials and Methods

### 2.1 Materials and Reagents

Various reagents, including HEPES, NaCl, CaCl_2_, NiCl_2_, KCl, MgCl_2_, 3-aminopropyltriethoxysilane (APTES), trimethylamine (TEA) and polyethylene glycol 8000 were purchased from Sigma-Aldrich. Dulbecco’s modified Eagle medium (DMEM) was obtained from Biochrom (Cambridge, United Kingdom). Phosphate buffered saline (PBS, pH 7.4), Fetal bovine serum (FBS), HAM’s F12 and penicillin-streptomycin were acquired from Gibco.

We utilized a SARS-CoV-2 strain, SARS-CoV-2/human/Czech Republic/951/2020, sourced from the National Institute of Public Health (Prague, Czech). The virus, produced in Vero E6 cells, was precipitated using polyethylene glycol (PEG) 8000 and then concentrated by centrifugation. After resuspension in NaCl-Tris-EDTA buffer (NTE) buffer, it was purified via tartrate gradient centrifugation.

### 2.2 Cell Culture

Vero E6 cells (ATCC CRL-1586), a common cell line used in virology studies, were cultured on 35 mm diameter plastic Petri dishes using DMEM supplemented with 10% FBS, 500 units/mL penicillin, and 100 μg/mL streptomycin. Prior to AFM measurements, we ensured cells occupied approximately 10–30% of the dish surface. The growth medium was subsequently discarded, and 1–2 mL of physiological HEPES buffer (consisting of 140 mM NaCl, 5 mM KCl, 1 mM MgCl_2,_ 1 mM CaCl_2_, and 10 mM HEPES, pH 7.4 adjusted with NaOH) was added.^12^

CHO-K1 cells (ATCC CCL61), a cell line from the ovary of an adult, female Chinese hamster, were grown on Petri dishes using 1:1 mixture of DMEM and HAM’s F12 (Gibco) containing 10% FBS (Gibco), 500 unit/mL penicillin and 100 μg/mL streptomycin (Gibco). For AFM experiments, cells were transitioned to a physiological HEPES buffer, consisting of 140 mM NaCl, 5 mM KCl, 1 mM MgCl_2_, 1 mM CaCl_2_, and 10 mM HEPES, adjusted to pH 7.4 using NaOH. All experiments were conducted at room temperature.^13^

### 2.3 UV-C Device

A modified portable UV-C device from Samsung Electronics Co., Ltd. (Suwon, Korea) was utilized, consisting of four light emitting diode (LED) modules, each with a wavelength of 275 nm and a power of 5 mW (Figure S1A). The device delivers UV-C irradiation at a rate of 1.1 mW/cm^2^ throughout the disinfection cycle. Irradiation was measured in water using a portable UV radiometer 7.1 (GenUV, Daejeon, Korea) positioned 2 cm away from the light source.

### 2.4 Ozone Gas Generator

We employed an ozone gas generator (Figure S1B), which was constructed with a dielectric barrier discharge plasma reactor and stainless-steel electrodes affixed to an alumina dielectric plate (Figure S1C).^14^ The perforated electrode, with a 3 mm pore size, uses an 8 kV sinusoidal voltage at 12.5 kHz to generate plasma. Our reactor features an upper chamber with valve ports for purging residual ozone gas. For safety, the generator was housed within a fume hood’s ventilation system. We measured ozone gas concentration using absorption spectroscopy at a wavelength of 253.65 nm with a mercury lamp.^7^

### 2.5 Inactivation of SARS-CoV-2 by UV-C and Ozone Gas Treatment

Recently isolated SARS-CoV-2 from cell culture, without beta-propiolactone inactivation, was treated with either UV-C or ozone gas for varying durations (0–30 min). For each treatment condition, four wells in 48-well plates were used, each containing a mica slide (1 cm × 1 cm) and 100 μL of virus (2 × 10^5^ plaque-forming units (PFU)/mL). Following each treatment, 300 μL of PBS was added to the wells to collect the virus for plaque assay.^15−17^ For control experiments, the virus suspension in 300 μL of PBS collected from untreated samples was used to infect Vero E6 cells at a multiplicity of infection (MOI) of 0.01. These cells, seeded at a density of 2 × 10^5^ cells per well in 12-well plates, were incubated overnight at 37 °C in a 5% CO_2_ atmosphere to prepare for the plaque assay. Similarly, the virus suspension in 300 μL of PBS collected from treated samples was used to infect the host cells under the same conditions. The survival rate of the virus was calculated as a percentage by comparing the viral titers of the treated samples to those of the untreated control.

### 2.6 Sample Preparation for Bio-AFM

For safety reasons, SARS-CoV-2 was inactivated using beta-propiolactone (1:2000 for 36 h), and the success of this process was confirmed by a plaque assay on Vero E6 cells prior to Bio-AFM analysis.^16,17^ Existing literature confirms that, despite some partial damage to the spike protein caused by inactivation with beta-propiolactone, a significant portion of the spike protein remains intact on the virus, as shown by TEM images.^18^ The inactivated SARS-CoV-2 (stock concentration: 8.85 log plaque forming unit (PFU)/ml) solution was diluted in a 1:20 ratio with imaging buffer (10 mM HEPES, 150 mM NaCl, 2 mM CaCl_2_, pH 7.4) containing 5 μM NiCl_2_. We placed 50 μL of the viral solution onto mica discs, incubated it for 3 min, and then rinsed it with approximately 100 μL of the imaging buffer without drying. Subsequently, the chamber was filled with 200 μL of imaging buffer. We then subjected these samples to UV-C or ozone gas treatments for 1, 3, 5, and 7 min each. After treatment, the sample was placed in the AFM imaging chamber for analysis.

### 2.7 Structural Analysis of SARS-CoV-2 Using Bio-AFM

To analyze virus structures, we employed a Bio-AFM (Agilent 5500, Agilent Technologies) in a fluid cell filled with 500 μL of imaging buffer supplemented with NiCl_2_. Magnetically coated AFM cantilevers (Type VII MAC Lever, NanoWorld), featuring a nominal spring constant of 0.1 N/m, facilitated imaging in magnetic AC (MAC) mode. We conducted meticulous AFM measurements opting for a resonance frequency between 9 and 11 kHz in liquid and maintaining a scan line speed of 1 Hz.^19^

Images were processed using Gwyddion 2.5565 software.^20^ Horizontal artifacts, stemming from feedback instabilities, were identified and mitigated utilizing Laplacian background substitution. A height threshold mask facilitated the selection of the background, establishing a baseline, and subsequently addressing scan line artifacts and polynomial background discrepancies. All experiments were replicated four times.

### 2.8 Cantilever Preparation for SVFS

In the experimental setup described, the process involved the functionalization and utilization of tipless cantilevers for SVFS to study the binding dynamics of the SARS-CoV-2 virus to host (Vero E6 cells) cells under various treatment conditions.

The preparation of the cantilevers (MLCT-O10, Bruker, CA) began with their amino-functionalization using APTES via a gas-phase method,^21^ ensuring a surface primed for further modification. In brief, the cantilevers were then pegylated using a custom-synthesized *N*-hydroxysuccinimide (NHS)-PEG-acetal linker in chloroform. The cantilevers were immersed overnight at 4 °C in a solution containing NaCNBH_3_ and inactivated SARS-CoV-2 virus, followed by thorough washing in PBS to ensure cleanliness and biocompatibility for the AFM measurements. This preparation is essential for maintaining the biological integrity of the samples during the measurement process. Prior to the measurements, each cantilever, now with the virus attached and featuring a spring constant of 0.01 N/m, was positioned within a chamber filled with 600 μL of imaging buffer. These virus-conjugated cantilevers, measured at room temperature, exhibited cantilever constants ranging from 6.7 to 9.7 pN/nm, derived from thermally driven oscillations.

The final phase of the preparation involves subjecting the samples to either UV-C radiation or ozone gas. These sterilization treatments were applied for specific durations of 3, 10, and 30 min, respectively. This step is designed to assess the effects of these disinfection methods on the structural integrity and functional capabilities of the virus particles attached to the cantilevers.

For control experiments on cantilevers without inactivated SARS-CoV-2 virus, the end was conjugated with the PEG linker in the above-mentioned method except virus conjugation. For control experiments on CHOk1 cells, the cantilever end was conjugated with inactivated SARS-CoV-2 virus via the PEG linker.

### 2.9 SVFS Measurement and Analysis

Force measurements were conducted on Vero E6 cells using a HEPES buffer enhanced with 50 μM nickel ions, where each of the six cells underwent at least 100 force–distance (FD) curve assessments. Similarly, ten CHOk1 cells were analyzed in the HEPES buffer environment, with each cell also subjected to a minimum of 100 FD curves to ensure consistency and reliability in our comparative analysis.

In an effort to specifically investigate the interactions of the spike protein, free ACE2 molecules were introduced to the measurement solution at a concentration of 19 nM, targeting four previously measured Vero E6 cells for a subsequent series of at least 100 FD curves each. This blocking experiment aimed to elucidate the role of ACE2 in virus-cell binding dynamics.

Data analysis was carried out using Matlab R2013a, focusing on the critical evaluation of the loading rate—calculated by the product of the pulling speed and the effective spring constant that encapsulates both the studied molecules’ characteristics and the AFM cantilever’s properties. Deflection sensitivity was determined by analyzing the slope of FD curves against a plastic substrate, ensuring that each cell location faced 100–200 FD cycles throughout the experiments. These cycles, rich in detail with thousands of data points each, had a force threshold set around 5 pN, providing a comprehensive overview of the force mechanics at play during the virus-cell interactions.

The unbinding event was identified through a local maximum analysis using a signal-to-noise threshold of 2. The binding activity was calculated as the fraction of curves that showed unbinding events.^22^

### 2.10 Statistical Analysis

All data are expressed as mean ± standard deviation. Comparison of mean values between the two groups was performed using analysis of variance (ANOVA) and Student’s *t*-test. Statistical significance levels were set as *p < 0.05, **p < 0.01, ***p < 0.001.

## 3 Results and Discussion

### 3.1 Attenuation of SARS-CoV-2 Infectivity via UV-C and Ozone Gas Treatments

**Figure 2:**
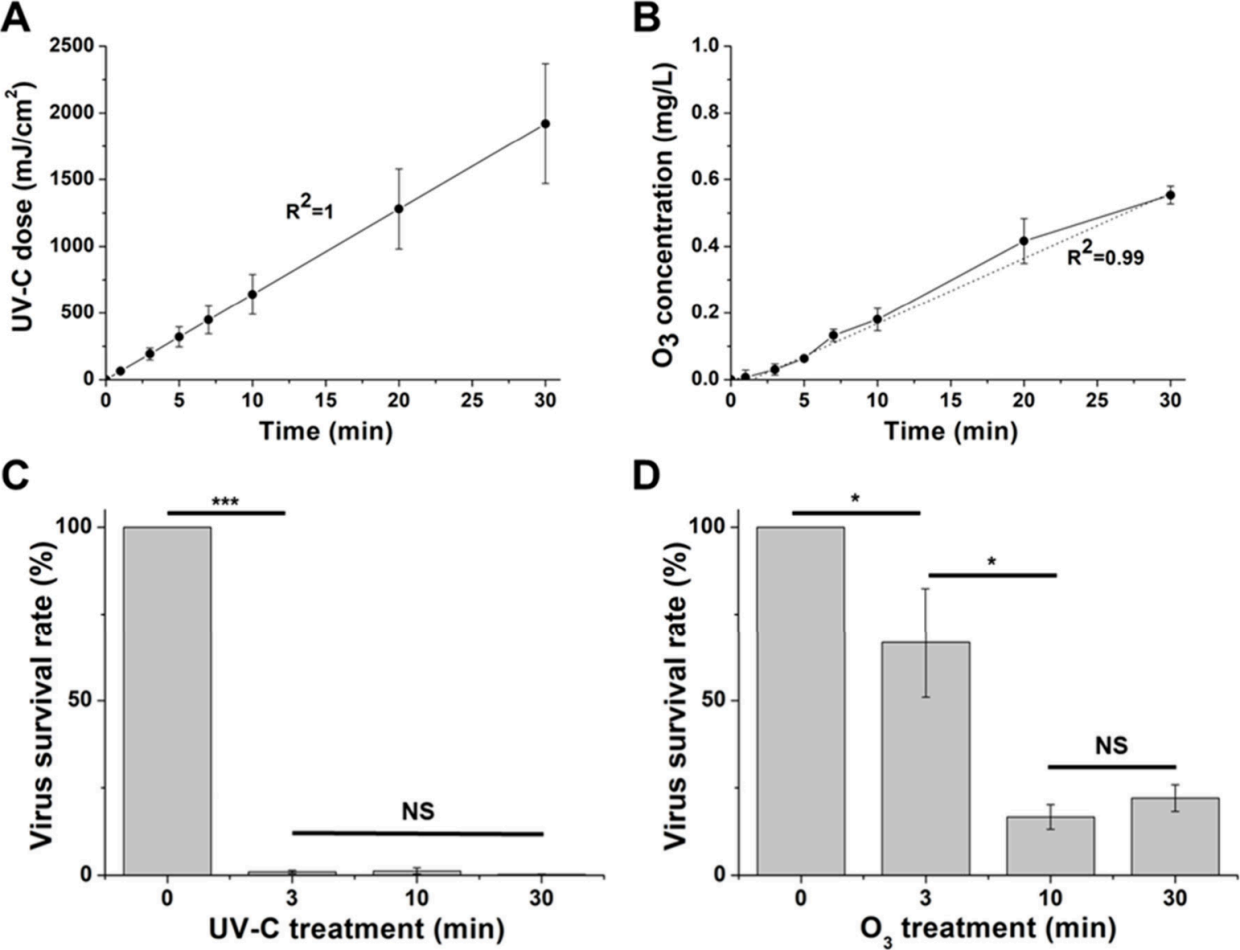
Measurements of UV-C dose (A) and ozone gas (O_3_) concentration (B) were taken across various treatment durations. The survival rates of SARS-CoV-2 under UV-C (C) and ozone gas (O_3_) (D) exposure were assessed at specific time intervals (3, 10, 30 min). Following the treatment with UV-C or ozone gas (O_3_) in 48-well plates, viral samples (initial concentration: 10^5^ PFU/mL) were collected and used to infect Vero E6 cells (*n* = 4). The infectivity was subsequently evaluated using plaque assays, and the virus survival rate was determined as a percentage. Mean values were statistically compared using Student’s *t* test, with *p < 0.05, ***p < 0.001 indicating significance and with NS representing nonsignificance. All experiments were conducted at biosafety level 3.

The dose of UV-C irradiation showed a linear progression with the duration of treatment, reaching levels of 64, 192, 320, 448, 640, 1280, and 1920 mJ/cm^2^ (R^2^ = 1, Figure 2A). Similarly, the ozone gas concentration in the imaging buffer increased to 0.007, 0.03, 0.063, 0.132, 0.18, 0.416, and 0.553 mg/L (R^2^ = 0.99, Figure 2B). Notably, the ozone gas began dissolving in the imaging buffer immediately upon injection into the chamber. The solubility of ozone gas was approximately 0.1, which is very close to the reported literature value of about 0.14 for NaCl solutions.^23^

Pertinently, UV-C treatment of SARS-CoV-2 resulted in a significant reduction of viral titer that consistently exceeded the 99% threshold (Figure 2C). Conversely, ozone gas treatment initially showed a relatively low infectivity reduction of around 33% post 3 min of exposure. Intriguingly, this reduction rate increased significantly to approximately 90% following 10 and 30 min of treatment (Figure 2D). This result is similar to previously reported findings, which showed a reduction of approximately 94.2% at an ozone concentration of 0.4 mg/L.^6^ The modest reduction may be due to the result of ozone gas concentrations increasing less rapidly than UV-C dose, which shows a milder sterilization potency. Additionally, the low solubility of ozone gas in the buffer may have resulted in a smaller amount of ozone actually affecting the virus. The absorption of ozone gas by plastics,^24^ a factor that could potentially reduce the effectiveness of ozone sterilization, prompted an investigation into the material-dependent efficacy of ozone gas treatment. To this end, we conducted experiments to compare sterilization outcomes on different substrates by inoculating both slide glass and plastic well plates with *Escherichia coli* O157:H7 (ATCC 43889) bacteria, followed by exposure to ozone gas. The findings indicated a stark contrast in sterilization effectiveness: while the slide glass showed no remaining bacterial presence, indicating complete eradication, the plastic well plates exhibited a significantly reduced sterilization efficiency (Figure S2). This discrepancy highlights the diminished effectiveness of ozone sterilization on plastic surfaces, presumably due to the material’s propensity to adsorb ozone gas, thereby attenuating the gas’s availability for microbial inactivation. This study underscores the importance of considering substrate materials in the application of ozone gas for sterilization purposes.

### 3.2 Impact of UV-C and Ozone Gas on Topographical Characteristics of SARS-CoV-2

**Figure 3:**
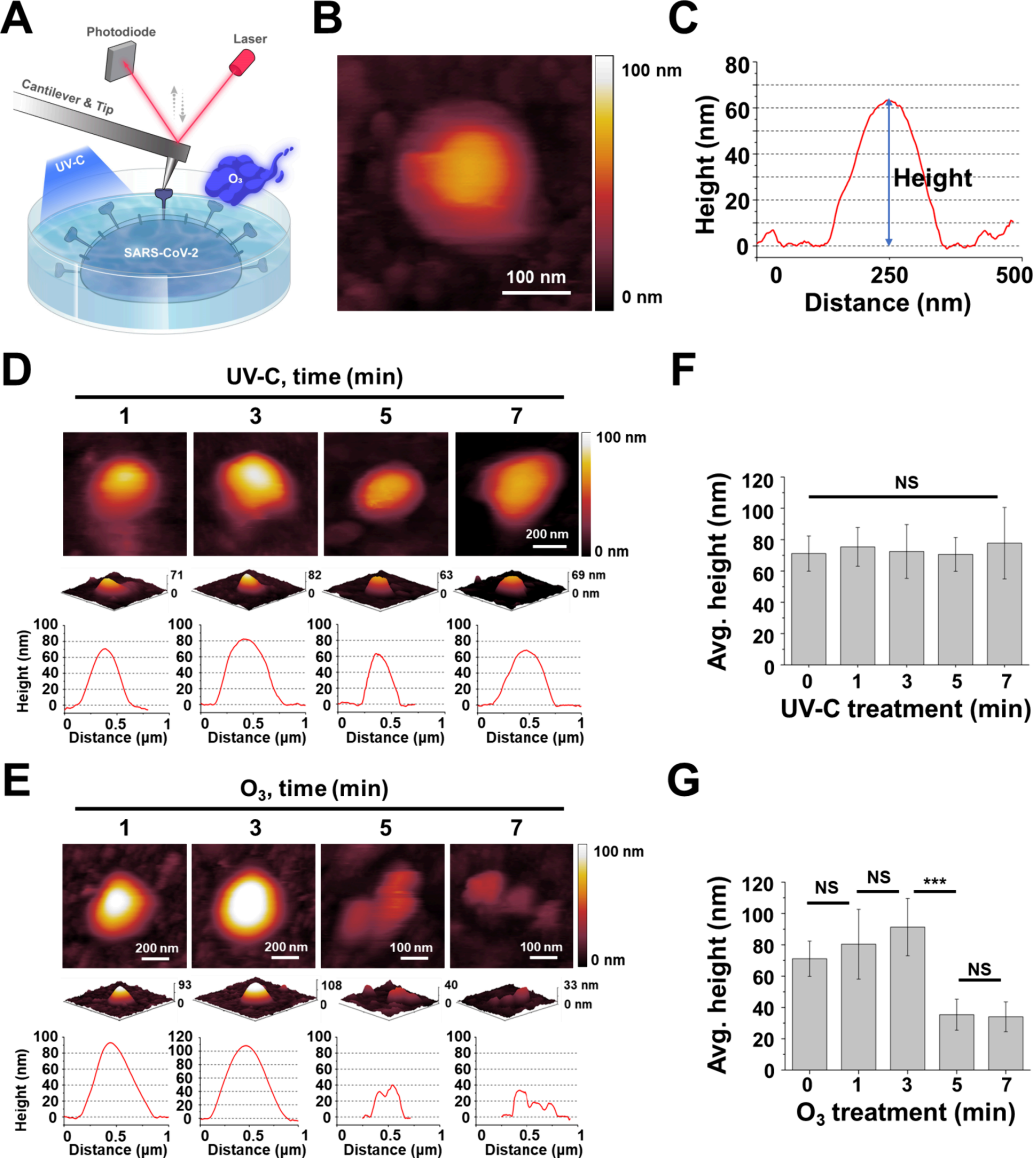
(A) Schematic representation of topographic imaging for virus analysis via biological atomic force microscopy (Bio-AFM), before and after treatment with UV-C radiation or ozone gas. Imaging was performed using a Bio-AFM (Agilent 5500, Agilent Technologies) within a fluid cell holding 500 μL of imaging buffer supplemented with NiCl_2_. The AFM utilized Type VII MAC Lever cantilevers (NanoWorld), which are coated with a magnetic layer and have a nominal spring constant of 0.1 N/m, facilitating imaging in MAC mode. (B–E) Bio-AFM imaging and analysis of SARS-CoV-2 particles on a mica surface. (B) showcases a Bio-AFM topographic image of an individual SARS-CoV-2 particle. (C) presents the corresponding height profile for the depicted virus particle. The impact of sterilization treatments on SARS-CoV-2 is illustrated through topographic images and height profiles after UV-C exposure (D) and ozone gas (O_3_) exposure (E) for durations of 1, 3, 5, and 7 min, respectively (*n* = 4 for each condition). (F) and (G) compare the average height of SARS-CoV-2 particles following treatment with UV-C and ozone gas (O3), respectively (*n* = 10 to 20). Error bars represent the standard deviation of the height measurements. Statistical analysis of mean values was performed using ANOVA, with significance levels denoted as ***p < 0.001 and with NS indicating nonsignificant differences.

Figure 3A depicts a Bio-AFM topographic image of inactivated SARS-CoV-2 immobilized in a nickel-enriched imaging buffer. By carefully exploiting the electrostatic interaction between the negatively charged mica surface and the positively charged nickel cations, inactivated SARS-CoV-2 was stably immobilized on the mica surface.^25^ During Bio-AFM imaging, multiple fragments were also observed on the mica surface, with prominent circular structure reflecting the typical morphological characteristics of the virus (Figure 3B, Figure S3). Although Bio-AFM stands out for its exceptional sensitivity along the *z*-axis, there is still the potential for subtle distortion of the width measurement of a spherical shape due to the tip convolution effects, resolution limitations which lead to an overestimation of lateral dimensions. In order to mitigate this effect, the full width at half-maximum (fwhm) method was used to analyze the diameter of the virus. The ensuing analysis of the height profiles of these demarcated structures revealed a mean width of 147.9 ± 40.1 nm, complemented by an average height of 71.1 ± 11.2 nm (n = 20) (Figure 3C) which then was considered as boundary criterion of a single virus particle for further analysis. To address potential concerns regarding the structural integrity of inactivated SARS-CoV-2, it is important to note that the inactivated virus retains its spherical structure, which is consistent with established benchmarks in the literature. Previous research has documented that the size of the virus typically ranges from 70 to 80 nm.^8,11,26^ Additionally, while the treatment with the organic solvent beta-propiolactone for virus inactivation may cause partial damage to the spike protein, a significant portion of the spike protein structure remains intact.^18^ Consistent with the literature, our observations, supported by high-speed atomic force microscopy (HS-AFM) videos (Videos S1 and S2), further demonstrate that the virus maintains its integrity, with proteins still diffusing on its surface. These findings collectively indicate that the inactivation process does not significantly compromise the structural appearance of SARS-CoV-2, ensuring that the virus remains a valid subject for detailed structural and functional analyses postinactivation.

### 3.3 Impact of UV-C and Ozone Gas on Structural Characteristics of SARS-CoV-2

To discern alterations in SARS-CoV-2 morphology post-treatment with UV-C and ozone gas under each treatment condition, we employed Bio-AFM for topographical analysis. Virus images from all topographical analyses represent different virus particles for each treatment condition. Morphological variations in inactivated SARS-CoV-2 were probed across incremental durations of UV-C exposure. After 1 min of UV-C irradiation, the spherical morphology of inactivated SARS-CoV-2 remained largely intact (Figure 3D) and was consistent with the control (Figure 3B). This persistent structural identity was retained even with irradiation increments to 3, 5, and 7 min, devoid of perceptible morphological shifts in the inactivated SARS-CoV-2 (Figure 3D, Figure S3).

In stark contrast to UV-C treatment, striking morphological adaptations were observed in inactivated SARS-CoV-2 subjected to elongated ozone gas treatment durations (Figure 3E, Figure S3). Following 1 and 3 min of ozone gas application, no significant morphological alterations were discerned. However, upon 5 min of exposure, the viral surface began to manifest roughness and indications of damage. The formerly spherical viral entities witnessed a degradation in their structural coherence, accompanying a diminished height and observable fragmentation. Extending ozone gas treatment to 7 min instigated further deterioration in viral architecture. We hypothesize that the substantial exposure to ozone gas inactivated a notable fraction of the virus, which was subsequently removed from the surface during washing, leaving behind the imaged viral remnants.

To quantitatively validate these morphological shifts, we contrasted the average height through a meticulous height profile analysis. Flat or lowest areas of the scanned surface were identified, and a height-zero baseline was established by utilizing software to flatten the image and calibrate these regions to a height of zero. When multiple peaks were observed in the height profile from the analysis, the height measurement was taken from the tallest peak. An initial investigation into the average height of inactivated SARS-CoV-2 post–UV-C irradiation across varied time points was undertaken (Figure 3F), revealing measurements of 75.4 ± 12.4 nm post 1 min, 72.5 ± 17.2 nm post 3 min, 70.6 ± 10.8 nm post 5 min, and 77.8 ± 22.8 nm post 7 min of UV-C exposure (Table S1). No statistically substantiated distinctions were evident across these measurements. Preceding research, deploying TEM, indicated the retention of viral morphology post–UV-C treatment.^5^ UV-C radiation, renowned for its ability to permeate viruses, catalyzes the formation of uracil dimers within viral RNA, undermining RNA replication and propelling viral incapacitation.^27^ Consequently, our observations underscore that UV-C irradiation negligibly impacted the virus’s structural height or shape, corroborating earlier findings.

In contrast, evaluating the average height of inactivated SARS-CoV-2 postozone gas treatment revealed divergent outcomes relative to UV-C (Figure 3G). Average heights post 1 min of ozone gas application measured at 80.4 ± 22.3 nm, marginally increasing to 91.3 ± 18.3 nm post 3 min. However, the height of the virus markedly plummeted to 35.4 ± 9.9 nm post 5 min and further shrunk to 34.1 ± 9.5 nm post 7 min of ozone gas application (Table S1). This sequential height reduction corroborates the theory that surface membrane damage triggers the egress of internal components, culminating in a decrease in height.^28^ Preceding studies have substantiated that ozone gas can inflict damage upon viral envelopes and cellular membranes.^9,29^ Additionally, when ozone gas was applied for 1 and 3 min, the size of the virus particles tended to increase. This is most likely due to the expanded membrane induced by ozone treatment. Understanding the biomechanical properties of viral membrane, including capsids, is critical for viruses to maintain their structural integrity under various conditions, which is critical for their ability to infect host cells.^11,30,31^ According to Nonn et al., Young’s modulus values are 20–30 MPa for the outer layer of the viral membrane and 8.5–9.0 MPa for the inner layer.^31^ The inner layer, which consists of hexagonally arranged ribonucleoproteins (RNPs) and water, is softer than the outer layer, which contains the M protein within the lipid bilayer.^32^ We assume that the ozone-induced damage can decrease the Young’s modulus of the outer layer, leading to swelling of the virus particles. A reduction in Young’s modulus of the outer layer may cause the RNPs inside to exert outward pressure, further expanding the membrane and resulting in particle swelling. These observations starkly juxtapose UV-C treatment, which imparted no alterations in viral height or morphology. Conversely, ozone gas treatment elicited surface damage, precipitating a notable height reduction and significant morphological alterations.

To understand the local surface roughness of virus particle affected by each treatment, the root-mean-square (RMS) roughness was measured on a 100 × 100 nm^2^ areas (n = 4) on each virus particle.^33^ Prior to any treatment, the virus demonstrated a roughness of 2.3 nm (Table S1). Across all UV-C treatment time conditions, roughness did not exhibit significant variation. In contrast, post 1 and 3 min of ozone gas treatment, roughness values lingered at 2.17 and 2.8 nm, respectively. Nevertheless, post 5 min of treatment, roughness escalated to 4.8 nm, and post 7 min, it sharply surged to 7.18 nm.

### 3.4 Impact of UV-C and Ozone Gas on Binding Activity between SARS-CoV-2 and Host Cells

**Figure 4:**
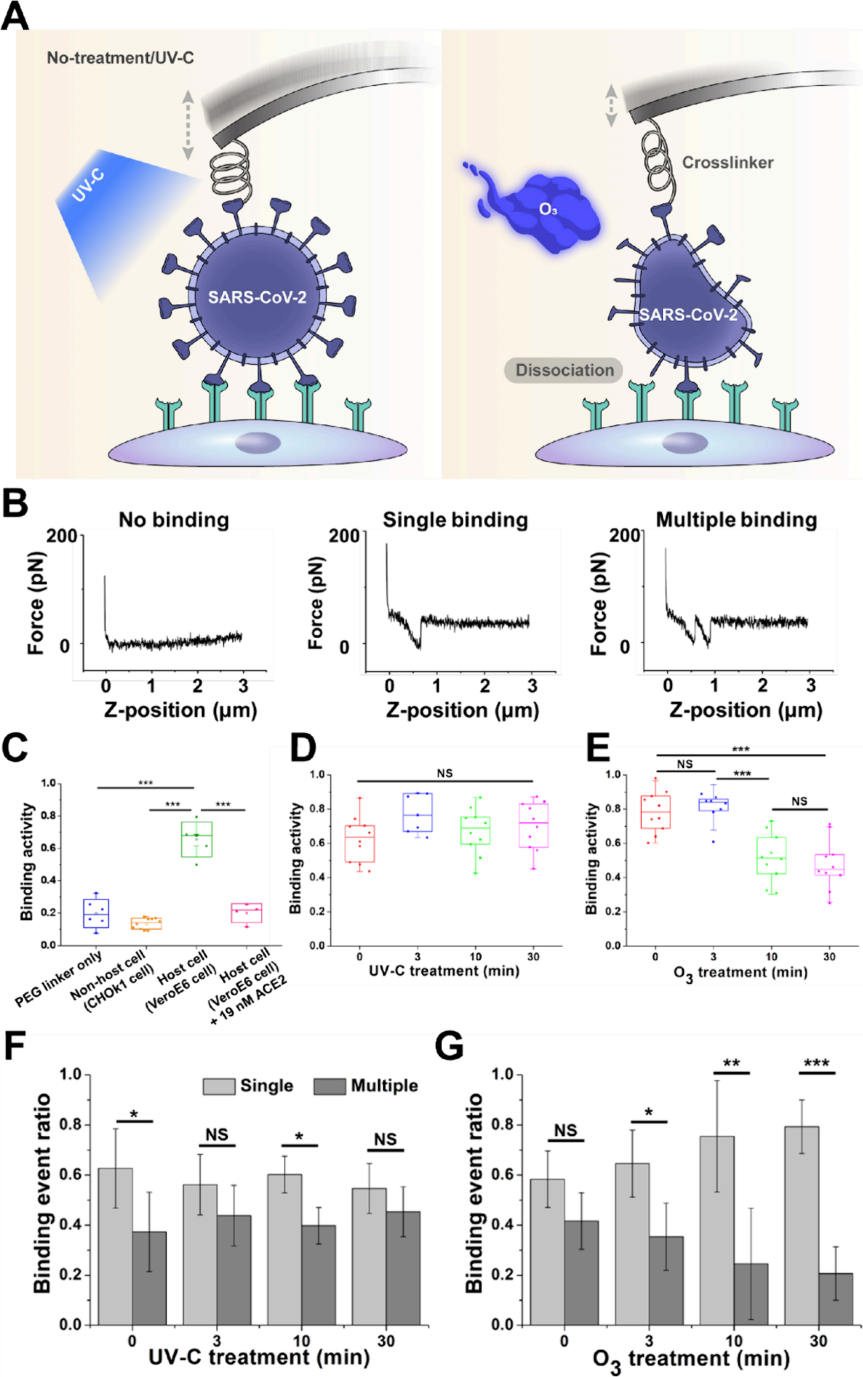
(A) Schematic representation of force measurement experiments between inactivated SARS-CoV-2 and Vero E6 cells, detailing conditions with and without exposure to UV-C or ozone gas treatments. (B) Example of a force–distance cycle showing specific cell–virus unbinding. (C) A cantilever devoid of the virus exhibited significantly reduced binding (blue dots), demonstrating minimal nonspecific interactions. Conversely, a cantilever functionalized with the virus displayed approximately 66% binding rate to Vero E6 cells (green dots), underscoring virus-specific interactions. Binding activity notably decreased to 15% (orange dots) when employing CHOk1 cells, which lack the ACE2 receptor. The introduction of free ACE2 molecules (final conc 19 nM) led to a reduction in binding to 20% (red dots). Binding activity between inactivated SARS-CoV-2 and Vero E6 cells after UV-C treatment (D) and after ozone gas (O_3_) treatment (E). Ratio of single to multiple binding between inactivated SARS-CoV-2 and Vero E6 cells after UV-C treatment (F) and after ozone gas (O_3_) treatment (G). In our SVFS analysis, we measured each point between 100 and 200 times across approximately 10 different locations within a single cell, resulting in over 1000 curves in total. Mean values were statistically compared using Student’s *t* test, with *p < 0.05, **p < 0.01, ***p < 0.001 indicating significance and with NS representing nonsignificance.

To elucidate the influence of UV-C and ozone gas treatments on the binding activity between inactivated SARS-CoV-2 and Vero E6 cells, a detailed analysis was executed employing virus-affixed cantilevers (Figure 4A). The virus was anchored to the cantilever through initial amino group functionalization, followed by the linkage via acetal-PEG-NHS linker.^21^ The subsequent treatments of these virus-coupled cantilevers with UV-C or ozone gas enabled the examination of any resultant alterations in binding activity. These treated cantilevers were then introduced to Vero E6 cells in over 100 interactions to quantify the binding activity. Employing a Bio-AFM, a minor force was applied to the sample surface, and the deflection of the cantilever was measured to validate virus binding. The interaction between the cantilever and the sample undergoes modifications upon virus binding to Vero E6 cells. This results in cantilever deflection, and the specific interaction force is detectable within the retract signal (Figure 4B). These signals were subjected to analysis to determine the extent of binding activity (defined as the percentage that showed a specific interaction force in FD curve).^34^ In our AFM setup, we emphasize the use of the tipless cantilever for precise single-virus contact with host cells for accurate measurements of virus-cell interactions. A specialized polymer linker attaches the virus to the cantilever, maintaining the virus’s structural integrity and biological activity during experiments. With this procedure, the cantilevers were sparsely covered with viruses (Figure S4). Therefore, with the given tilting angle of the cantilever (∼20°) during virus force spectroscopy experiments, the probability is very low that two or more viruses touch the surface at the same time with the applied moderate forces (about 100 pN). This combination of a finely tuned cantilever operation and a biocompatible linker allows for targeted manipulation of single virus particles, ensuring that the observed interactions closely represent the natural binding events between the virus and host cell receptors. Additionally, the method we used for attaching the virus to the cantilever, involving specific tip chemistry, produced an F-D curve comparable to those found in other single virus force spectroscopy studies,^34−36^ with forces comparable to the isolated spike protein.^22^

Before examining the effects of UV-C or ozone treatments on the binding behavior of the inactivated SARS-CoV-2 virus, control experiments were conducted to establish the specificity of the virus’s interactions with host cells. These controls involved comparisons with situations where only a PEG linker was used, nonhost cells were engaged, and host cells were exposed to 19 nM ACE2 (as depicted in Figure 4C and Figure S5). The binding activity between the host cells and the inactivated SARS-CoV-2 virus-conjugated cantilever was significantly higher than in any control group (*p* < 0.001%). Introducing free ACE2 molecules at a concentration of 19 nM into the assay notably reduced binding activity to 20%, highlighting the critical role of ACE2 in these interactions. These findings affirm that the binding events between the inactivated SARS-CoV-2 virus and Vero E6 cells are highly specific to the ACE2 receptor, emphasizing the precision of these viral interactions.

The untreated virus illustrated a robust binding activity of approximately 63%. Intriguingly, the UV-C treated samples did not demonstrate a statistically significant alteration in binding activity: 77% at 3 min, 67% at 10 min, and 70% at 30 min (Figure 4D). Given the previously discussed lack of UV-C impact on viral morphology, it is plausible that the inherent structural integrity of the spike protein, pivotal for binding, remains unscathed, thus, sustaining the binding activity. Contrastingly, ozone gas treatment unveiled a nuanced pattern in binding activity: an initial elevation to 80% post 3 min, succeeded by a conspicuous decline to 51% and 47% after 10 and 30 min respectively (Figure 4E). This reduction suggests that ozone gas initiates the degradation of surface proteins, leading to diminished binding activity. While there are several factors involved in the binding of SARS-CoV-2 to cells, the surface proteins, particularly the spike protein, are crucial for binding to the ACE2 receptor on Vero E6 cells.^21^ Therefore, any alteration in the structural integrity of these proteins could potentially compromise their binding activity. However, if ozone treatment does not completely disintegrate the virus or only partially modifies specific viral proteins or the membrane, it may lead to scenarios where the altered viral particles can still attach to cell surfaces. Nonetheless, this binding would likely be nonspecific and nonfunctional. Although the virus might attach to the cell, the essential steps required for viral entry and replication would be hindered due to the oxidative damage inflicted by ozone.

When we further analyzed the ratio of single to multiple binding between the virus-attached cantilever and Vero E6 cells, we noted notable differences following interventions with UV-C and ozone gas. Interestingly, the ratio of single to multiple binding remained largely unchanged for the UV-C treated virus, even with prolonged treatment duration (Figure 4F, Figure S6A). This constancy suggests that UV-C treatment may not significantly impact the spike protein of the virus, aligning with our prior discussions. In contrast, ozone gas treatment exhibited a distinct effect on the ratio of single to multiple binding, with multiple binding predominating as a control and post 3 min of treatment, and single binding becoming more prominent post 10 min of treatment. As indicated in topographic data (Figure 3E), disruption of the viral membrane reduces the effective contact area for interaction with the cell surface, resulting in lower binding activity and a decreased likelihood of multiple binding (Figure 4G, Figure S6B). The observed increase in the ratio of single binding could be attributed to ozone gas-induced damage to the surface proteins, particularly the spike protein, resulting in fewer available binding sites on the virus.

## 4 Conclusions

The exploration into the sterilization effects of UV-C and ozone gas on SARS-CoV-2 unveiled distinct outcomes concerning viral inactivation and structural integrity. UV-C treatment displayed a steadfast capability to inactivate SARS-CoV-2, achieving an impressive sterilization rate of over 99% across various treatment durations (3–30 min) while preserving the virus’s morphological stability. This efficacy supports its ability to obstruct viral replication by penetrating the virus and inhibiting its RNA without inducing structural changes. On the other hand, ozone gas, although demonstrating a somewhat diminished effectiveness at 3 min, managed to secure approximately 90% sterilization after a 10 min exposure, albeit at the expense of inducing apparent morphological alterations and virus particle damage upon extended exposure. Binding activity investigations, utilizing AFM technology and cantilever-based measurements, revealed that while UV-C minimally affected binding activity, presumably by preserving surface proteins essential for binding, ozone gas led to a decrease in binding activity correlated with increased exposure duration, likely due to its disruptive action on surface proteins. In summary, UV-C emerges as a potent and consistent anti-SARS-CoV-2 agent, adeptly balancing sterilization with structural preservation, while ozone gas, although capable of sterilization, impairs viral integrity and binding activity, particularly at higher concentrations and durations. These insights pave the way for refining and expanding current sterilization and preventive strategies against SARS-CoV-2, providing a scientific foundation for further research and practical applications in viral mitigation.

## Supporting Information Available

Optical images of the UV-C device and the ozone gas (O_3_) generator components; infectivity test results for *E. coli* O157:H7 on slide glass and 48-well plates; Bio-AFM images of SARS-CoV-2 particles on a mica surface following UV-C and ozone gas (O_3_) treatments.; Bio-AFM images of SARS-CoV-2 particles on silicon surface utilizing the same cantilever surface chemistry as for virus force spectroscopy; example of force–distance curves; force probability density function (PDF) from inactivated SARS-CoV-2 on Vero E6 after UV-C treatment and after ozone gas treatment; average height of SARS-CoV-2 particle surface after UV-C or ozone gas (O_3_) treatment for 1, 3, 5, and 7 min (*n* = 4), and root-mean-square (RMS) roughness of SARS-CoV-2 particle surface after UV-C or ozone gas (O_3_) treatment for 1, 3, 5, and 7 min; HS-AFM setup (PDF)
HS-AFM Video S1 of the SARS-CoV-2 particle on mica surface (MP4)
HS-AFM Video S2 of the SARS-CoV-2 particle on mica surface (MP4)
