# Supplementary material for: Mechanisms of Plasma Ozone and UV-C Sterilization of SARS-CoV-2 Explored through Atomic Force Microscopy
Source: ACS Appl Mater Interfaces. 2024 Sep 6;16(37):49176–85. doi: 10.1021/acsami.4c11057 (PMC11420863; doi:10.1021/acsami.4c11057)
Supplement: Supplementary file 1 — am4c11057_si_001.pdf [file am4c11057_si_001.pdf]

Supporting Information

Mechanisms of Plasma Ozone and UV-C Sterilization of SARS-CoV-2 Explored through Atomic Force Microscopy

Jinseung Bae[1], Petr Bednar[2,3,4], Rong Zhu[5], Cheolwoo Bong[1], Moon Soo Bak[1], Sarah Stainer[5], Kyoungjun Kim[6], Junghun Lee[6], Chulsoo Yoon[6], Yugyeong Lee[7], Omobolaji Taye Ojowa[5], Maximilian Lehner[5], Peter Hinterdorfer[5], Daniel Ruzek[2,4,8], Sungsu Park[1,7], Yoo Jin Oh[5]**

[1]School of Mechanical Engineering, Sungkyunkwan University (SKKU), Suwon, 16419, Republic of Korea

[2]Veterinary Research Institute, CZ-62100 Brno, Czech Republic

[3]Department of Medical Biology, Faculty of Science, University of South Bohemia, CZ-37005 Ceske Budejovice, Czech Republic

[4]Department of Experimental Biology, Faculty of Science, Masaryk University, CZ-62500 Brno, Czech Republic

[5]Institute of Biophysics, Johannes Kepler University Linz, Linz, A-4020, Austria

[6]Samsung Electronics, Suwon, 16677, Republic of Korea

[7]Department of Biomedical Engineering, Sungkyunkwan University (SKKU), Suwon, 16419, Republic of Korea

[8]Institute of Parasitology, Biology Centre of the Czech Academy of Sciences, CZ-370 05 Ceske Budejovice, Czech Republic

*Email: nanopark@skku.edu

*Email: Yoo_Jin.Oh@jku.at

KEYWORDS: Sterilization mechanisms; Infectivity test; Topographical characteristics; Structural characteristics; Binding activity

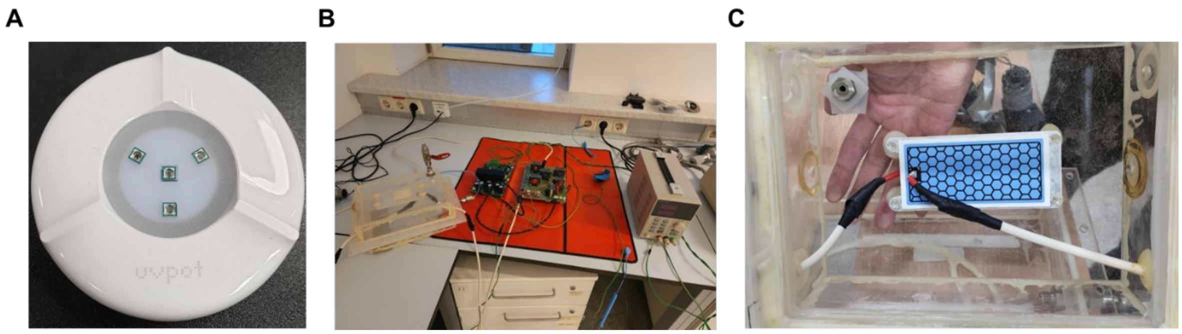

Figure S1. Optical images showcase the UV-C device (A) and the ozone gas (O_3) generator components (B, C). (A) The UV-C device contains four LED modules, each with a 275 nm wavelength and 5 mW power output. (B) The generator is equipped with an acrylic chamber, measuring 23 x 17 x 12 cm (W x L x H), and includes a high-voltage, high-frequency power supply. (C) Within this chamber, a specialized electrode for ozone gas (O_3) production is installed.

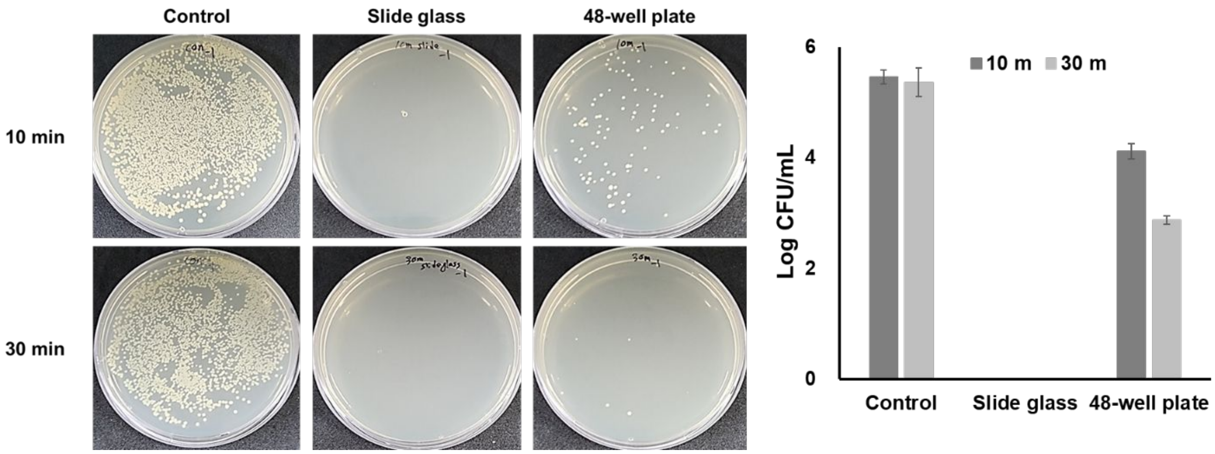

Figure S2. Infectivity test results for *E. coli* O157:H7 on slide glass and 48-well plates: (A) optical images of the plates used for colony counting. (B) log reduction of E. coli O157:H7 on slide glass and 48-well plates (n=3). For the experiments, 100 μL of a bacterial solution (10^6 CFU/mL) was deposited onto mica slides (1 cm × 1 cm) placed either in 48-well plates or on slide glass, and then immediately exposed to ozone gas for 10 or 30 minutes, respectively. The ozone gas concentrations measured on the slide glass were 0.28 mg/L for 10 minutes and 1 mg/L for 30 minutes, while those in the 48-well plate were 0.18 mg/L for 10 minutes and 0.55 mg/L for 30 minutes. Following the ozone treatment, 300 μL of PBS was added to the wells to collect the bacteria for colony counting.

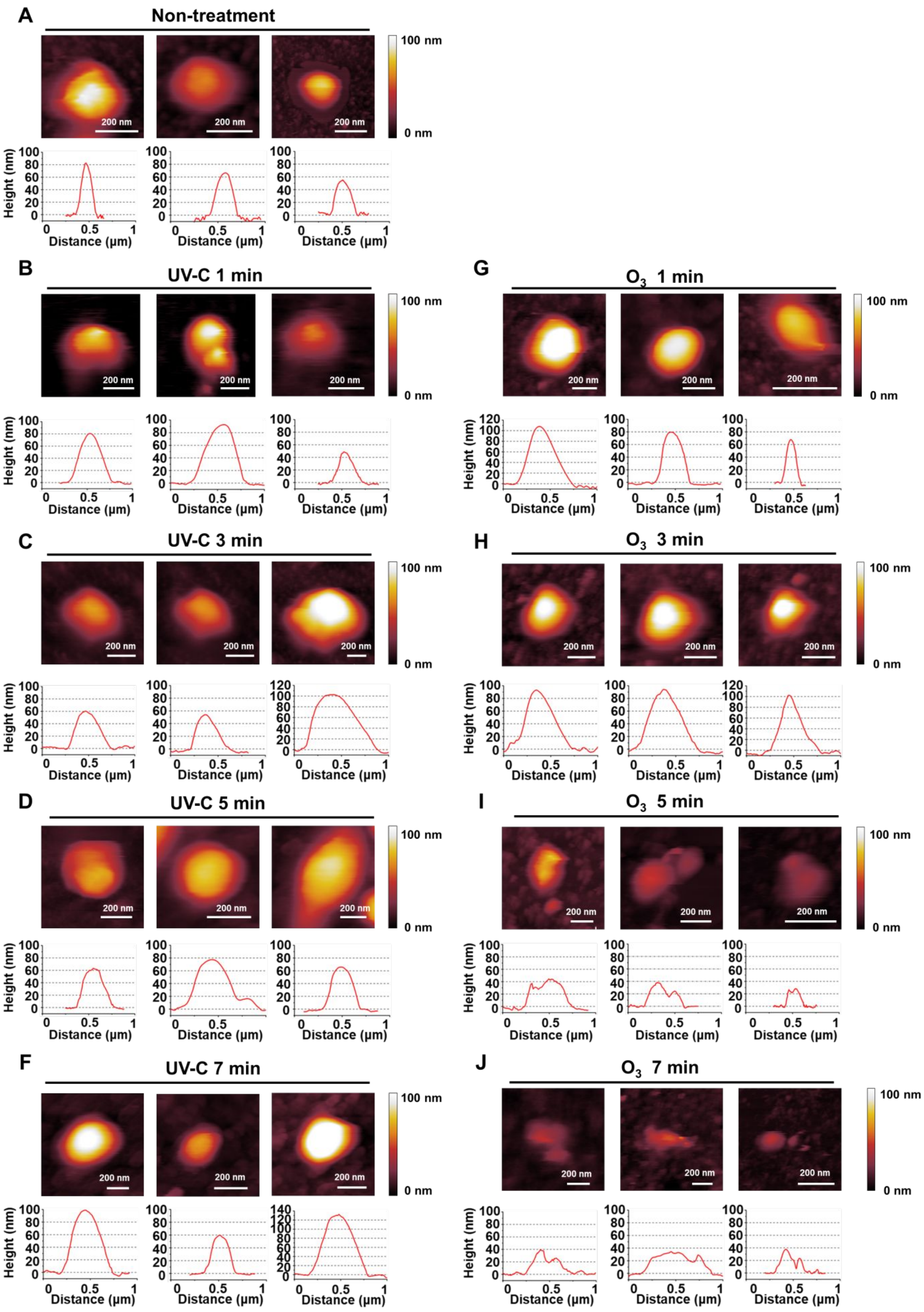

A
Non-treatment
B
UV-C 1 min
C
UV-C 3 min
D
UV-C 5 min
F
UV-C 7 min
G
O_3 1 min
H
O_3 3 min
I
O_3 5 min
J
O_3 7 min
200 nm
100 nm
0 nm
Height (nm)
Distance (µm)

Figure S3. Bio-AFM images of SARS-CoV-2 particles on a mica surface following UV-C and ozone gas (O_3) treatments. Panel (A) displays images of three different virus particles that have not been treated with either UV-C or ozone gas. Panels (B) through (E) display the effects of UV-C treatment at 1, 3, 5, and 7 minutes, respectively, while panels (G) through (J) show the effects of ozone gas treatment for the same intervals. In each treatment group, four distinct virus particles were analyzed. This figure illustrates the topographic images of three different particles per condition, while images of the fourth particle are presented in Fig. 3D and 3E. Height profiles and line analyses were conducted on each particle to quantify morphological changes and maintain consistent measurement standards across all samples. These analyses are crucial for supporting the height variation data shown in Fig. 3F and 3G.

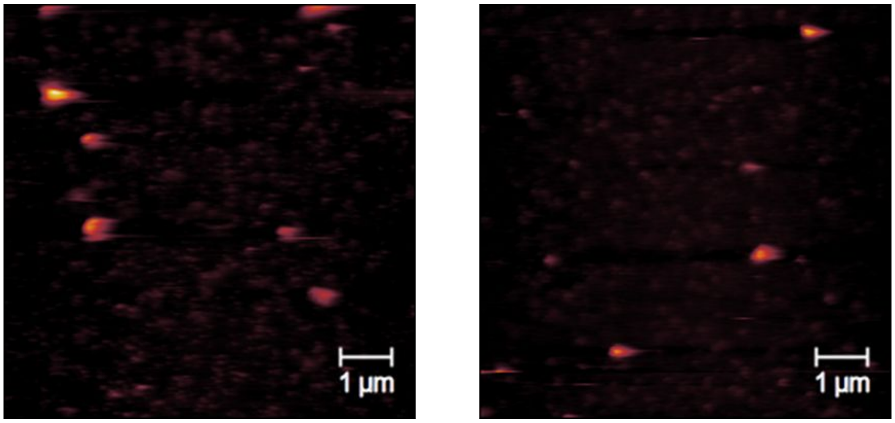

Figure S4. Bio-AFM images of SARS-CoV-2 particles on silicon surface utilizing the same cantilever surface chemistry as for virus force spectroscopy.

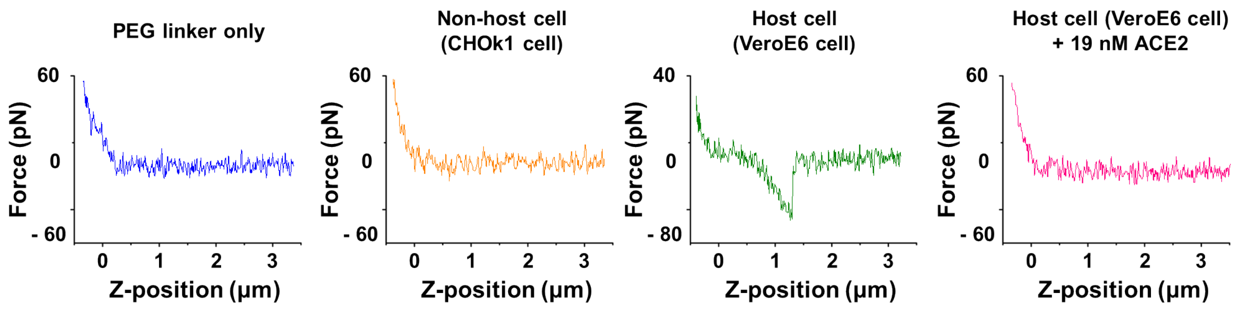

Figure S5. Example of force-distance curves. (A) Tip with PEG linker only but without inactivated SARS-CoV-2 virus, on VeroE6 cell. (B) Tip with inactivated SARS-CoV-2 virus, on CHOk1 cell. (C) Tip with inactivated SARS-CoV-2 virus, on VeroE6 cell, before block. (D) Tip with inactivated SARS-CoV-2 virus, on VeroE6 cell, after block with 19 nM ACE2.

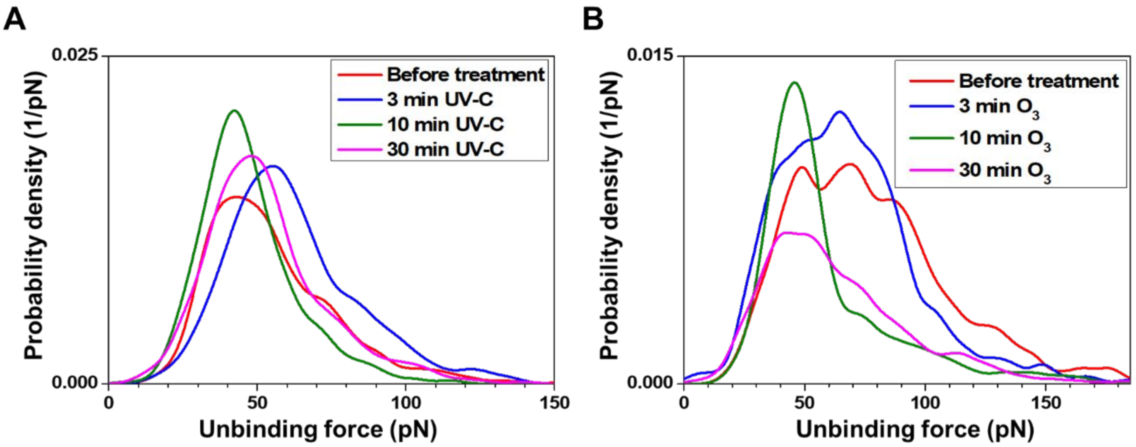

Figure S6. Force probability density function (PDF) from inactivated SARS-CoV-2 on VeroE6 after UV-C treatment (A) and after ozone gas (O_3) treatment (B) were quantified using atomic force microscopy (AFM; Agilent 5500, Agilent Technologies). These force distributions were represented as probability density functions of dissociation forces, which indicate the binding multiplicity through the local maxima of the curves. The results suggest that UV-C treatment does not seem to cause a reduction in multiple bindings, whereas ozone gas (O_3) treatment gradually reduces multiplicity, especially at 10 and 30 minutes.

UV-C (min)	Average height ± SD (nm)	RMS roughness (nm)
0	71.1 ± 11.2	2.3
1	75.4 ± 12.4	1.6
3	72.5 ± 17.2	2.7
5	70.6 ± 10.8	2.34
7	77.8 ± 22.8	2.76

O_3 (min)	Average height ± SD (nm)	RMS roughness (nm)
0	71.1 ± 11.2	2.3
1	80.4 ± 22.3	2.17
3	91.3 ± 18.3	2.8
5	35.4 ± 9.9	4.8
7	34.1 ± 9.5	7.18

Table S1. Average height of SARS-CoV-2 particle surface after UV-C or ozone gas (O_3) treatment for 1, 3, 5, and 7 min (n=10-20), and root mean square (RMS) roughness of SARS-CoV-2 particle surface after UV-C or ozone gas (O_3) treatment for 1, 3, 5, and 7 min.

UV absorption spectroscopy. The ozone concentration was measured using ultraviolet absorption spectroscopy. Light from a mercury lamp (BHK 90-0005-01, spectral line: 253.65 nm) was directed through a lens and optical fiber, then passed through a cuvette containing a buffer in which the ozone was dissolved. The intensity of the transmitted light was measured using a spectrometer (AvaSpec-2048L, Avantes, Apeldoorn, The Netherlands). Given that the absorption cross section of ozone is broad near 253.65 nm and known to be 1.137×10^{-17} cm^2/molecule, the ozone concentration was calculated using the Beer-Lambert law, as described in Equation (1) below:

$$c = \frac{Absorbance}{\varepsilon l} \quad (1)$$

Absorbance is defined as -log(I/I_0), where I and I_0 represent the light intensities of the ozone-treated sample and the non-treated sample, respectively. In this context, ε is the ozone molar absorption coefficient, which is 2900 M^{-1} cm^{-1}, and l is the optical path length, set at 1 cm in the region of interest.[1] The measurements were repeated three time.

HS-AFM setup. HS-AFM (RIBM, Ibaraki, Japan) was employed to obtain the topography of SARS-CoV-2 after UV-C and ozone treatments. We used an ultra-short cantilever USC-F1.2-k0.15 (NanoWorld, Neuchâtel, Switzerland) with a nominal spring constant of 0.15 N/m, a resonance frequency approximating 500 kHz, and a quality factor close to 2 in a liquid environment.

During imaging acquisition, the amplitude was stably maintained and set to 85-90% of the free amplitude, correlating to roughly 3 nm.

Video S1. HS-AFM video of the SARS-CoV-2 particle on mica surface.

Video S2. HS-AFM video of the SARS-CoV-2 particle on mica surface.

REFERENCES

(1) Hart, E. J.; Sehested, K.; Holoman, J. Molar absorptivities of ultraviolet and visible bands of ozone in aqueous solutions. *Anal. Chem.* 1983, 55 (1), 46-49.
